# Crystal structure of a cold-active protease (Pro21717) from the psychrophilic bacterium, *Pseudoalteromonas arctica* PAMC 21717, at 1.4 Å resolution: Structural adaptations to cold and functional analysis of a laundry detergent enzyme

**DOI:** 10.1371/journal.pone.0191740

**Published:** 2018-02-21

**Authors:** Ha Ju Park, Chang Woo Lee, Dockyu Kim, Hackwon Do, Se Jong Han, Jung Eun Kim, Bon-Hun Koo, Jun Hyuck Lee, Joung Han Yim

**Affiliations:** 1 Division of Polar Life Sciences, Korea Polar Research Institute, Incheon, Republic of Korea; 2 Unit of Polar Genomics, Korea Polar Research Institute, Incheon, Republic of Korea; 3 Department of Polar Sciences, University of Science and Technology, Incheon, Republic of Korea; 4 Department of Pharmacy, Graduate School, Sungkyunkwan University, Suwon, Republic of Korea; 5 Aniscien Co., Ltd., Jeonju, Republic of Korea; Russian Academy of Medical Sciences, RUSSIAN FEDERATION

## Abstract

Enzymes isolated from organisms found in cold habitats generally exhibit higher catalytic activity at low temperatures than their mesophilic homologs and are therefore known as cold-active enzymes. Cold-active proteases are very useful in a variety of biotechnological applications, particularly as active ingredients in laundry and dishwashing detergents, where they provide strong protein-degrading activity in cold water. We identified a cold-active protease (Pro21717) from a psychrophilic bacterium, *Pseudoalteromonas arctica* PAMC 21717, and determined the crystal structure of its catalytic domain (CD) at a resolution of 1.4 Å. The Pro21717-CD structure shows a conserved subtilisin-like fold with a typical catalytic triad (Asp185, His244, and Ser425) and contains four calcium ions and three disulfide bonds. Interestingly, we observed an unexpected electron density at the substrate-binding site from a co-purified peptide. Although the sequence of this peptide is unknown, analysis of the peptide-complexed structure nonetheless provides some indication of the substrate recognition and binding mode of Pro21717. Moreover, various parameters, including a wide substrate pocket size, an abundant active-site loop content, and a flexible structure provide potential explanations for the cold-adapted properties of Pro21717. In conclusion, this is first structural characterization of a cold-adapted subtilisin-like protease, and these findings provide a structural and functional basis for industrial applications of Pro21717 as a cold-active laundry or dishwashing detergent enzyme.

## Introduction

In recent years, the interest in cold-active (psychrophilic) enzymes as potential candidates for biotechnological applications has grown [1]. At low temperatures, psychrophilic enzymes from cold-adapted microorganisms have higher catalytic efficiencies than their mesophilic counterparts [2]. Psychrophilic proteases in particular have the potential for commercial use, especially as laundry detergent enzymes owing to their higher activities during washing processes that take place in cold tap water (below 15°C). Alkaline proteases are known to have significant activity and stability at high basic pHs, such as within a pH range of 10–12, and in the presence of surfactants and oxidizing agents [3, 4]. Therefore, psychrophilic alkaline proteases have attracted much interest from industrial enzyme manufacturers for inclusion in new laundry detergent formulations because of their higher cold-activity and stability under harsh washing conditions. To date, however, the activities of only a few psychrophilic proteases have been characterized at low temperatures [5–8]. Thus, contrary to our expectations, limited studies have been performed to characterize the performance of psychrophilic proteases as additives in laundry and dishwashing detergents.

Previously, we identified *Pseudoalteromonas arctica* PAMC 21717 from the Polar and Alpine Microbial Collection (PAMC) as a microbe of interest because it secretes a high level of a psychrophilic alkaline serine protease [9, 10]. Subsequently, we partially purified the protease (Pro21717) from PAMC 21717 and characterized the properties that could make it useful as a new additive enzyme for laundry detergents, including low-temperature activity, structural flexibility with broad substrate specificity, and stability under alkaline and high-surfactant conditions.

In most cases, subtilisin-like serine proteases are synthesized as enzymatically inactive zymogens, the activation of which invariably requires one or more proteolytic cleavages of the precursor [11]. In addition, it has been shown that the prosequence sometimes acts as a specific inhibitor, thus preventing unwanted proteolytic activity in the cytoplasm [12]. Pro21717 is a multi-domain protease consisting of a signal peptide, a prosequence, a catalytic domain (CD), and two pre-peptidase C-terminal domains (PD1 and PD2). The secreted mature Pro21717 protein contains no PD domains, indicating that the PD domains are removed during secretion and they may not be essential for the enzymatic activity. Pro21717 may degrade organic material found in seawater (the natural habitat of PAMC 21717) for use as an energy source. Homology searches using BLAST indicate the presence of Pro21717 homologs in other species. BLAST results show that the amino acid sequence of Pro21717 is highly conserved and similar to the corresponding proteins from other species, including *Rheinheimera nanhaiensis* (GenBank Accession No. GAB57675; 64% identity and 75% similarity), *Alishewanella aestuarii* (EJI84718; 62% identity and 73% similarity), and *Microbulbifer agarilyticus* (AQQ69198; 54% identity and 69% similarity).

Here, we describe the crystal structure of the catalytic domain of Pro21717 at 1.4 Å resolution. In this study, residues 27–483 of Pro21717, corresponding to the prosequence and catalytic domains (Pro21717-PCD), were expressed in *Escherichia coli* as a fusion protein with a C-terminal histidine tag and purified under denaturing conditions. During refolding of the precursor protein, an active protease (Pro21717-CD, residues 145–483) was produced by intramolecular cleavage of the precursor. The high-resolution crystallographic characterization of Pro21717-CD in the co-purified, substrate-bound state reveals how a peptide substrate can be accommodated within the catalytic domain. Moreover, the structural features of the Pro21717-CD were analyzed in terms of their suitability for cold adaptation. Thus, we succeeded in our aim of deriving the structure for Pro21717 to gain insight into its catalytic mechanism, which could be used for protein engineering in commercial applications, for example, in laundry and dishwashing detergents with improved cold-water performance.

## Materials and methods

### Library construction and screening for proteolytic clones

A genomic DNA library of Antarctic *Pseudoalteromonas arctica* PAMC 21717 was constructed using a CopyControl Fosmid Library Production Kit (Epicentre), as recommended by the manufacturer. Recombinant pCC1FOS vectors containing 35–45 kb DNA fragments were transformed into competent *E*. *coli* EPI300 cells. Fosmid clones were spread on LB plates supplemented with chloramphenicol (12.5 μg/mL), skim milk (1.2%), and CopyControl fosmid autoinduction solution (1×). After a 48-h incubation at 25°C, a proteolytic fosmid clone (EPI-P38) was detected by the presence of a clear halo around the colony. The recombinant fosmid was isolated and digested with eight different restriction enzymes that recognize the various restriction sites present in the destination vector (the high-copy plasmid pUC19). The DNA fragments from each restriction reaction were mixed, purified, and ligated into the pUC19 vector, after it was linearized with the same restriction enzymes and dephosphorylated. The ligation reaction was transformed into competent *E*. *coli* Rosetta cells. The plasmid clones were screened for protease activity on LB plates supplemented with carbenicillin (100 μg/mL) and skim milk (1.2%). After a 48-h incubation at 25°C, a plasmid clone (Rosetta-P38-4), producing a clear halo, was selected based on its proteolytic activity.

The inserted DNA of the recombinant proteolytic plasmid clone was completely sequenced by primer walking from both ends of the pUC19 cloning site, and the nucleotide sequences were assembled using DNAstar Lasergene software (DNAstar, USA). Similarity analysis of the sequences was performed using the BLAST program to search against the GenBank database. Protein domains were predicted using the Conserved Domain Database at NCBI.

### Plasmid construction for functional analysis of the psychrophilic serine protease gene

To prepare recombinant proteins for determining proteolytic activities and domain functions, the full open reading frame of the *pro21717* gene and various domain regions were PCR amplified from PAMC 21717 genomic DNA, cloned into the pEXP5-CT/TOPO over-expression vector (Invitrogen), and then transformed into competent *E*. *coli* BL21star(DE3)pLysS cells (Invitrogen). A primer set specific for each plasmid construct was designed based on the corresponding DNA sequence: Pro21717 construct, 5′-ATGACAACAAGTAAAACTTTTAAAAGATGCGC-3′ (forward) and 5′- CACTTAGCGGACAATACCAACCG-3′ (reverse); Pro21717-PCD construct, 5′- ATGCAATCAGTTTCAAGTTCAATGGC-3′ (forward) and 5′-GCAGCTGTTGCAGCAGCAAGT-3′ (reverse). PCR amplification was performed using the Biometra T3000 Thermal Cycler (Labrepco Inc., USA). PCR was performed in 100-μL reaction mixtures containing approximately 500 ng of PAMC 21717 genomic DNA, 50 pmol of each primer, and MG Taq-HF DNA polymerase (Macrogen, Korea). The thermocycling conditions used were: 5 min hot-start denaturation (94°C); 35 cycles of denaturation at 94°C for 30 s, annealing (approximately 5°C below T_m_ of the primers) for 45 s, and extension at 72°C for approximately 1 min/kb DNA’ and a final extension at 72°C for 5 min.

### Production and functional analysis of recombinant protease domains

*E*. *coli* BL21star(DE3)pLysS cells were transformed with recombinant plasmids encoding Pro21717-PCD or the Pro21717-CD domain. One milliliter of transformed *E*. *coli* cells grown in LB medium was transferred to 50 mL of LB/carbenicillin (100 μg/mL) and cultured to an optical density at 600 nm (OD_600_) of 1.0 at 37°C. Following cooling to 15°C for 30 min, the culture was induced by adding 1.0 mM isopropyl β-D-1-thiogalactopyranoside (IPTG), with further incubation at 15°C for 72 h. After centrifugation, the culture supernatant was concentrated 50-fold using a Vivaspin column (10 kDa molecular-weight cutoff, Sartorius), and 14 μg of the concentrated supernatant was loaded on a zymogram gel containing 0.5% skim milk. The cell pellet was disrupted by sonication in 2.0 mL of 50 mM sodium phosphate buffer (pH 7.6) and centrifuged (4,520 × *g*, 30 min, 4 °C). The resulting insoluble fraction was analyzed by sodium dodecyl sulfate-polyacrylamide gel electrophoresis (SDS-PAGE).

### Expression, refolding, and purification of Pro21717-CD

*E*. *coli* BL21star(DE3)pLysS cells transformed with the Pro21717-PCD construct were grown at 37°C in 1 L of LB medium supplemented with ampicillin (100 μg/mL). After growing to an OD_600_ of 1.0, expression was induced with 1.0 mM IPTG at 37°C for 24 h. The cell pellet was obtained by centrifugation (10,000 × *g*, 30 min, 4°C), resuspended in 4 mL standard buffer (50 mM sodium phosphate, pH 7.6), and sonicated. After centrifugation (10,000 × *g*, 30 min, 4°C), the resulting insoluble fraction was washed twice (10,000 × *g*, 10 min, 4°C) with 20 mL of 0.5% Triton X-100. The washed inclusion body (1 g) was unfolded in 20 mL unfolding buffer (pH 8.5; 8 M urea, 50 mM mercaptoethanol, and 20 mM Tris-HCl) at 37°C for 1 h. The unfolded protein solution was diluted in 60 mL refolding buffer (20 mM Tris-HCl, 100 mM NaCl, 20 mM CaCl_2_, and 0.05% Tween 20) and subsequently dialyzed in 1.6 L refolding buffer at 4°C for 2–3 days. The refolded protein solution was loaded onto a Sephacryl S-100 column (1.6 mm × 70 cm, GE Healthcare) in a Dual Flow FPLC instrument (Bio-Rad). The proteins were separated according to size in the mobile phase (standard buffer) at a flow rate of 0.7 mL/min. A fraction with significant proteolytic activity was concentrated and used as a homogenous enzyme solution (designated as Pro21717-CD) in subsequent experiments for biochemical characterization and crystallization.

### Preliminary fermentation experiment for over-expression of Pro21717-CD

Cultivation of *E*. *coli* BL21star(DE3)pLysS cells transformed with the Pro21717-PCD construct was performed in a 5-L jar fermenter (Minifors, Infors HT, Switzerland) with an initial culture volume of 2 L. One hundred milliliters of seed-containing LB medium (10 g/L bactotryptone, 5 g/L yeast extract, 10 g/L NaCl) was prepared and incubated with shaking at 37°C, 200 rpm for 20 h. The fermentation medium used was modified R medium consisting of (NH_4_)_2_HPO_4_ (4.0 g/L), KH_2_PO_4_ (13.5 g/L), citric acid (1.7 g/L), bactotryptone (10.0 g/L), yeast extract (20.0 g/L), lactose (20.0 g/L), glycerol (50.0 g/L), MgSO_4_•7H_2_O (1.2 g/L), and 10 mL/L of a trace metal solution (FeSO_4_•7H_2_O [10.0 g/L], CaCl_2_•2H_2_O [2.0 g/L], ZnSO_4_•7H_2_O [2.25 g/L], MnSO_4_•4–5H_2_O [0.5 g/L], CuSO_4_•5H_2_O [1.0 g/L], (NH_4_)_6_Mo_7_O_24_•7H_2_O [0.1 g/L], Na_2_B_4_O_7_•10H_2_O [0.23 g/L], HCl [35%; 5.0 mL/L) [13]. The temperature was maintained at 37°C, and the pH was controlled from 6.8 to 7.0 using 14% (v/v) NH_4_OH. Cells were grown for 6.3 h after inoculation until the cell concentration reached 4 g dry cell weight (DCW)/L, and then IPTG was added to the medium at a final concentration of 1 mM to induce protease expression. Cell growth was monitored by measuring DCWs and the OD_600_ with a spectrophotometer (S-3150, Scinco, Seoul, Korea). The ratio of the DCW (g/L) to the optical density was 0.27. Protease activity assay was performed as described below.

### Crystallization of Pro21717-CD

Purified Pro21717-CD was concentrated to approximately 250 mg/mL using an Amicon Ultra-15 centrifuge filter (Millipore, Bedford, MA, USA) with a 10-kDa molecular-mass cutoff. Commercially available screening kits from Microlytic (MCSG1-4), Qiagen (Classic I&II), and Emerald Bio (Classic Wizard I-IV; Bainbridge Island, WA) were used for initial crystallization experiments, where 0.8 μL of a reservoir solution was added to 0.8 μL of protein solution in the drop of a sitting-drop 96-well crystallization plate (Emerald Bio) at 293 K using the Mosquito crystallization robot (TTP Labtech, Cambridge, MA). Crystals appeared spontaneously within 2 days. Pro21717-CD crystallized in a cluster of large needles from 0.16 M magnesium chloride, 0.08 M Tris-HCl buffer pH 8.5, 24% PEG 4000, and 20% glycerol (MCSG-1 #22 condition) at 293 K.

### Data collection and structure determination of Pro21717-CD

A single needle-shaped crystal was harvested with a cryoloop directly from the 96-well plate without further refinement of the crystallization conditions and was cryoprotected using paratone oil from a liquid-nitrogen gas stream. A 1.40 Å resolution data set containing 360 images was collected at the 7A beam line of the Pohang Accelerator Laboratory (PAL; Pohang, Korea) and diffraction images were indexed, integrated, and scaled using the HKL-2000 program [14]. The crystal of Pro21717-CD belongs to the *P*2_1_2_1_2_1_ space group and showed unit cell parameters of *a* = 47.95 Å, *b* = 74.56 Å, *c* = 83.01 Å, and *α = β = γ* = 90°. The subtilisin-like protein AprV2 (PDB code 3LPA) was used as a model for structure determination by molecular replacement using the program MOLREP [15]. The resulting coordinate was refined against the original data set using the REFMAC5 program, and the COOT program [16] was used for model building. After successive cycles of rebuilding and refinement, the *R*_work_ and *R*_free_ values were determined to be 0.132 and 0.156, respectively. Water molecules and calcium ions were added to the final model using the COOT program. The final model contains residues 145–483 of chain A, three co-purified alanine residues of chain B, four calcium ions, and 595 water molecules. The structures have 96.4% residues in favored regions of the Ramachandran plot [17] with no outliers. Data collection and refinement statics are given in Table 1. Molecular figures were generated using the PyMOL Molecular Graphics System Version 1.4 [18]. The atomic coordinates and structural factors of the Pro21717-CD (PDB code 5YL7) crystal structure have been deposited in the Protein Data Bank (http://www.rcsb.org/).

**Table 1:** X-ray diffraction data collection and refinement statistics.

**Data set**	
X-ray source	Beamline 7A, PAL
Space group	*P*2_1_2_1_2_1_
Wavelength (Å)	0.97934
Resolution range (Å)	30.00–1.40 (1.42–1.40)
No. of observed reflections	804441
No. of unique reflections	58245 (2849)
*R*_sym_ ^a^ (%)	6.6 (15.0)
Average *I/σ*	69.0 (36.3)
Completeness (%)	98.0 (98.2)
Multiplicity	13.8 (14.3)
**Refinement**	
Resolution (Å)	50.01–1.40 (1.44–1.40)
No. of reflections in working set	55253 (4010)
No. of reflections in test set	2939 (207)
No. of residues	338
No. of water molecules	595
No. of calcium ions	4
*R*_cryst_ ^b^ total (%)	13.15 (13.8)
*R*_free_ ^c^ total (%)	15.61 (15.8)
R.m.s. bond length (Å)	0.024
R.m.s. bond angle (°)	2.147
Average B value (Å^2^) (protein)	8.526

^a^*R*_sym_ = ∑|<*I*>—*I*|/∑<*I*>

^b^*R*_cryst_ = ∑||*Fo*|—|*Fc*||/∑|*Fo*|

^c^*R*_free_ was calculated with 10% of all reflections excluded from the refinement stages, using high resolution data.

Values in parentheses refer to the highest resolution shells.

### Enzyme assays

To determine the proteolytic activity, 1.1 mL of 50 mM sodium phosphate buffer (pH 7.6; standard buffer) containing 10 μg of protein was mixed with 0.65% (w/v) of the substrate azocasein. After incubating the reaction mixture at 30°C for 30 min, 0.9 mL of 110 mM trichloroacetic acid was added and the mixture was further incubated at 37°C for 30 min. To remove precipitant, the mixture was centrifuged (12,000 × *g* for 3 min at 4°C) and filtered through a 0.45-μm filter. The filtrate (0.5 mL) was mixed with 0.25 ml Folin & Ciocalteu’s phenol reagent and 1.25 mL of 500 mM sodium carbonate solution. After 30 min at 37°C, the OD_660_ was determined and the enzyme activity was calculated based on an l-tyrosine standard curve. One unit of protease activity was defined as the activity required to produce 1 nmol of tyrosine equivalents released amino acids per min, per mg of protein at 30°C.

### Effects of temperature and pH on Pro21717-CD

The optimal temperature for proteolytic activity was determined by incubating the enzyme reaction mixtures at different temperatures (0–70°C). The reaction mixture, consisting of 0.65% azocasein and 10 μg of Pro21717-CD in 0.99 mL standard buffer, was incubated for 30 min at each temperature. Subtilisin Carlsberg (6 μg) was used as a control enzyme. In addition, the denaturation temperature (T_m_) of Pro21717-CD was measured using circular dichroism (CD) spectroscopy. The signal at 220 nm was recorded while the temperature was increased from 5 to 95°C at intervals of 2.5°C.

The optimum pH for proteolytic activity was measured in a 1.1-mL reaction mixture containing 0.65% azocasein and 10 μg of Pro21717-CD after incubation at 30°C for 30 min. The pH was adjusted by using 50 mM sodium acetate (pH 4.0–6.0) or 50 mM potassium phosphate (pH 6.0–9.5). To examine the pH stability, 100 μg of Pro21717-CD was pre-incubated on ice for 1 h in 0.9 mL different buffers with various pH ranges, including 50 mM sodium acetate (pH 2.0–6.0), 50 mM potassium phosphate (pH 7.0–10.0), and 50 mM sodium tetraborate (pH 11.0). One hundred microliters of the pre-incubated Pro21717-CD (10 μg) was added to 1.0 mL standard buffer containing 0.65% azocasein and incubated at 30°C for 30 min. Subtilisin Carlsberg (6 μg) was used as a control enzyme at 40°C.

### Effects of metal ions and detergents on Pro21717-CD

To examine the detrimental effects of metal ions and other components of laundry detergents, 10 μg of Pro21717-CD was mixed with 1 mM CaCl_2_, CuSO_4_, NaCl, N_2_SO_4_, sodium linear alkylbenzene sulfonate (LAS), SDS, phenylmethylsulfonyl fluoride, or 1% H_2_O_2_ in 0.99 mL standard buffer, and incubated for 1 h on ice. Subsequently, 90 μL of 6.5% azocasein was added to the reaction mixture, and then the residual protease activity of Pro21717-CD was measured under the same conditions as described above.

### Substrate specificity of Pro21717-CD

To analyze the substrate specificity of Pro21717-CD, the ability to hydrolyze four kinds of macro proteins and seven different small synthetic peptides was measured. Proteolytic activities for the macro proteins collagen, keratin, skim milk, and azocasein (final concentrations, 0.65%) were measured based on the quantities of released amino acids in enzyme assays, as described above. Substrate specificity for the following synthetic substrates was tested by Peek’s method [19]: *N*-succinyl-Ala-Ala-Val-*p*-nitroanilide (AAV), *N*-succinyl-Ala-Ala-Pro-Leu-*p*-nitroanilide (AAPL), *N*-succinyl-Ala-Ala-Ala-*p*-nitroanilide (AAA), *N*-succinyl-Gly-Gly-Phe-*p*-nitroanilide (GGF), *N*-succinyl-Thr-Leu-Val-*p*-nitroanilide (TLV), *N*-succinyl-Ala-Ala-Pro-Phe-*p*-nitroanilide (AAPF), and *N*-succinyl-Ala-Ala-Val-Ala-*p*-nitroanilide (AAVA), which were purchased from Sigma—Aldrich Chemicals. Pro21717-CD (10 μg) was added to 750 μL standard buffer containing each substrate (final concentration, 0.83 mM) and incubated at 30°C for 5 min. To terminate the assay, 250 μL of 2 M sodium acetate (pH 5.0) was added and the absorbance was determined at 410 nm. The enzyme activity was calculated based on a *p*-nitroanilide standard curve. One unit of protease activity was defined as the amount of enzyme required to produce 1 mmol of released *p*-nitroanilide per min, per mg of protein at 30°C. Subtilisin Carlsberg was assayed under the same conditions as Pro21717-CD, except that the reaction temperature was increased to 55°C.

### Washing test

Pro21717-CD (20 μg) was incubated in 1.0 mL standard buffer containing 0.5, 1.0, or 5.0% of skim milk for 4 min at room temperature. Application of Pro21717-CD as a detergent additive was also examined on white cotton cloth (8 × 8 cm, Testfabrics Inc.) soiled with blood, milk, and ink. The cloth pieces were submerged in a laboratory flask containing Pro21717-CD (13 mg) and 500 mL of a standard formulation for LAS detergents, provided from a laundry detergent manufacturer (LG Household & Health Care, Korea), and shaken at low speed for 1 h at 15°C. After shaking, the cloth pieces were rinsed with tap water and dried.

## Results

### Screening of a library for proteolytic clones and sequence analysis of the protease gene Pro21717

A genomic library from PAMC 21717, consisting of approximately 1.2 × 10^5^ fosmid clones, was screened for clear halo production around colonies on LB plates supplemented with skim milk. From this library, 13 fosmid clones were selected for their high proteolytic activity. Among them, one positive clone (EPI-P38) was used to construct a small-insert DNA library using the high-copy plasmid pUC19, followed by further screening for clear halo production. A positive clone (Rosetta-P38-4) was selected from two clones with high proteolytic activity. The 4,909-bp insert carried by Rosetta-P38-4 was completely sequenced and a putative protease gene was detected. The protease gene, designated *pro21717* (GenBank accession number MG720561), was composed of a 2,127-bp sequence encoding 708 amino acids, having a calculated molecular weight of approximately 72.6 kDa. The N-terminal sequence of Pro21717, a mature psychrophilic protease secreted from *Pseudoalteromonas arctica* PAMC 21717, was determined to be GAQNSSWH, which perfectly matches the predicted amino acid sequence of the N-terminal region of the sequenced gene. The predicted amino acid sequence derived from the *pro21717* nucleotide sequence was analyzed using the NCBI protein–protein BLAST and CD-Search programs. The Pro21717 protein sequence showed 96–99% and 88% identity with serine protease precursors composed of 708 and 711 amino acids, respectively, from different *Pseudoalteromonas* sp. strains that are also found in cold seawater (deep sea and polar sea). CD-Search was used to identify the catalytic domain to either the Peptidase S8 family or the serine endo- and exo-peptidase clans of subtilases containing three conserved amino acid residues (Asp185, His244, and Ser425) that constitute a catalytic triad like that found in trypsin-like proteases [20].

To obtain more information regarding the Pro21717 functional domains, its sequence was aligned with two well-characterized cold-active proteases, PRO-2127 (GenBank Accession No. ADH04492) from *Pseudoalteromonas* sp. QI-1 [21] and MCP-03 (ABD92880) from *Pseudoalteromonas* sp. SM9913 [22]. These homologs have a four-domain composition that includes a signal peptide for enzyme secretion, an N-terminal pro-sequence for correct folding, a catalytic domain, and two bacterial pre-peptidase C-terminal domains for enzyme thermostability.

**Fig 1:**
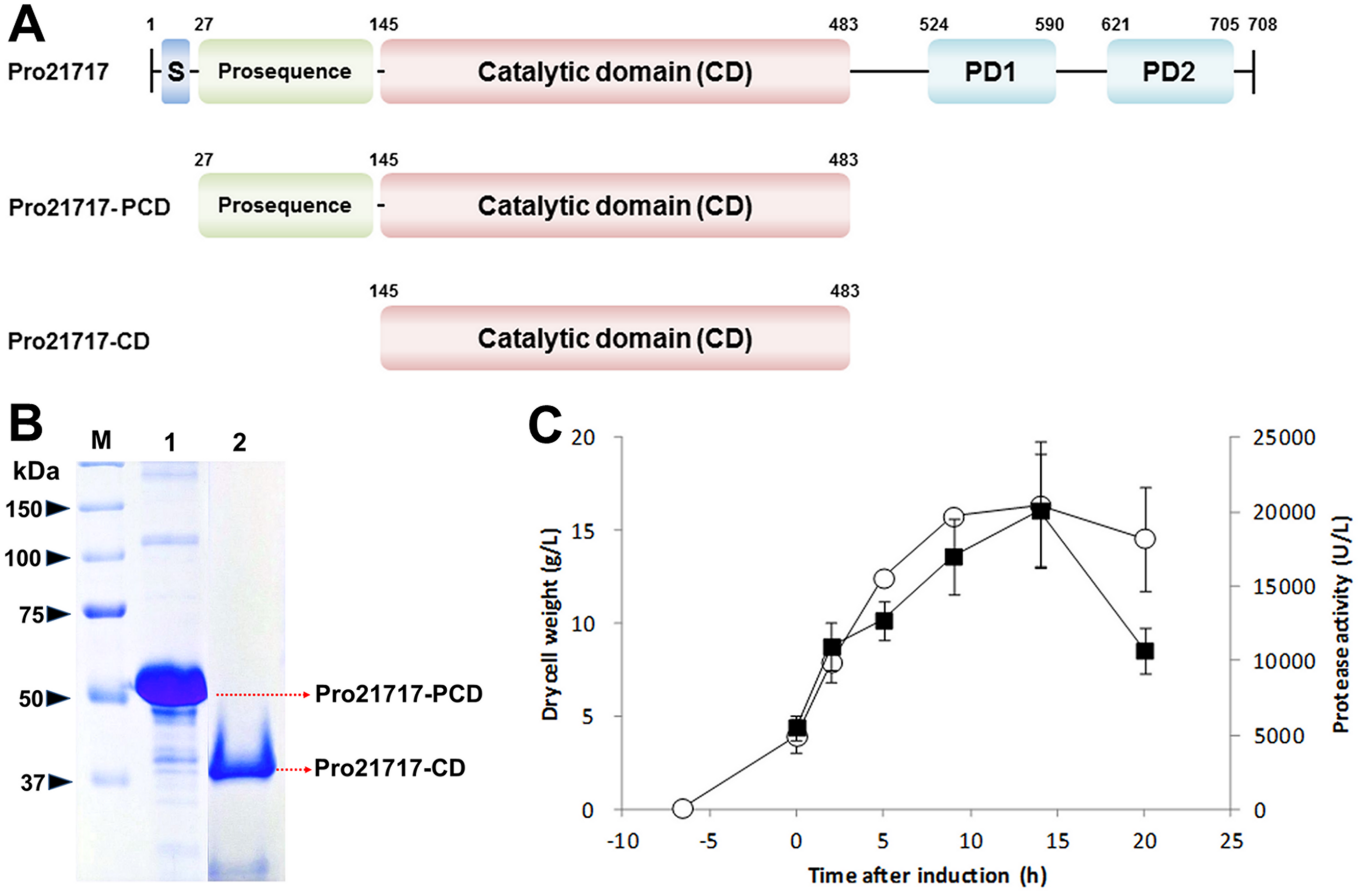
Expression and refolding of Pro21717-PCD. (A) Schematic diagrams of the Pro21717 domain structures. Pro21717 includes a precursor sequence (expressed from a 2,127-bp coding sequence) composed of four domains: a signal peptide, an N-terminal pro-sequence, a catalytic domain (CD), and two pre-peptidase C-terminal domains (PD1 and PD2). Pro21717 activation requires self-cleavage via its own catalytic domain to unmask the N-terminal pro-sequence. (B) SDS-PAGE analysis of the recombinant Pro21717 protease. Lane M, protein size marker; lane 1, Pro21717-PCD in inclusion bodies after washing; lane 2, self-cleaved Pro21717-CD after 48 h of refolding and purification through gel-filtration chromatography. (C) Time courses of cell densities (open circle) and protease activities (black square). Cells were grown in a 5-L jar fermenter using 2 L of modified R medium, with IPTG induction at a DCW of 4.

Preliminarily, the domain structure of the translated *pro21717* gene was predicted based on the results of the sequence alignments and secondary structure predictions. This analysis showed that the Pro21717 protease precursor consists of four functional domains (Fig 1A): a signal peptide (residues 1–27), an N-terminal prosequence (residues 28–142), a catalytic domain (residues 145–483), and two β-sandwich domains (residues 489–593 and 611–708). Most subtilase family members are synthesized as protease precursors, which are subsequently translocated across a cell membrane via the signal peptide and finally matured into an active form by cleavage of the signal peptide and the pro-sequence. Although subtilase precursors are usually mosaic proteins, the secreted mature form is usually composed of a single domain in a low-molecular weight range of 15–30 kDa [3, 22, 23]. Considering the structural properties and maturation processes of related subtilases, the 72.6-kDa precursor derived from *pro21717* may undergo a similar maturation process to generate an active form that could be restricted to the 34-kDa catalytic domain alone.

### Over-expression and purification of Pro21717-CD

*Escherichia coli* BL21star(DE3)pLysS cells expressing Pro21717-PCD were cultured and induced for over-production of the functional recombinant protease, consisting of the catalytic domain and pro-sequence region (a putative intramolecular chaperone). The overproduced recombinant protein was detected within the cell pellet in the form of inclusion bodies, rather than in the supernatant in soluble form. As described above (see Materials and methods), the washed inclusion bodies (1.0 g) were unfolded in unfolding buffer and dialyzed in refolding buffer, inducing spontaneous refolding and activation. Interestingly, during the refolding process, a shift in the size of the recombinant protease was detected on an SDS-PAGE gel: from approximately 50 kDa to 37 kDa after a 48-h incubation in the refolding buffer (Fig 1B). After allowing the refolding process to proceed for 72 h, approximately 240 mg of proteolytic protease was recovered with a specific activity of 116.4 ± 2.7 U/mg. Subsequently, the refolded protease was desalted and further purified by size-exclusion chromatography, resulting in a yield of 216 mg of homogeneous protease (designated Pro21717-CD) with a specific activity of 268.6 ± 7.5 U/mg. The average recovery rate from three independent experiments was estimated to be 22%, which is a relatively high yield at the laboratory scale (personal communications with experts).

A preliminary fermentation for protease over-production was performed using a bioreactor and modified R medium, and cell-density measurements were taken during the growth process. The cell density gradually increased and peaked at 16 g DCW/L after 14 h of induction, after which it slightly decreased to 15 g/L after 20 h of induction. The protease activity of refolded Pro21717-CD also varied with the induction time, reaching a maximum of 20,000 U/L after 14 h of induction and then decreasing to 11,000 U/L after 20 h of induction (Fig 1C).

### Overall structure of Pro21717-CD

**Fig 2:**
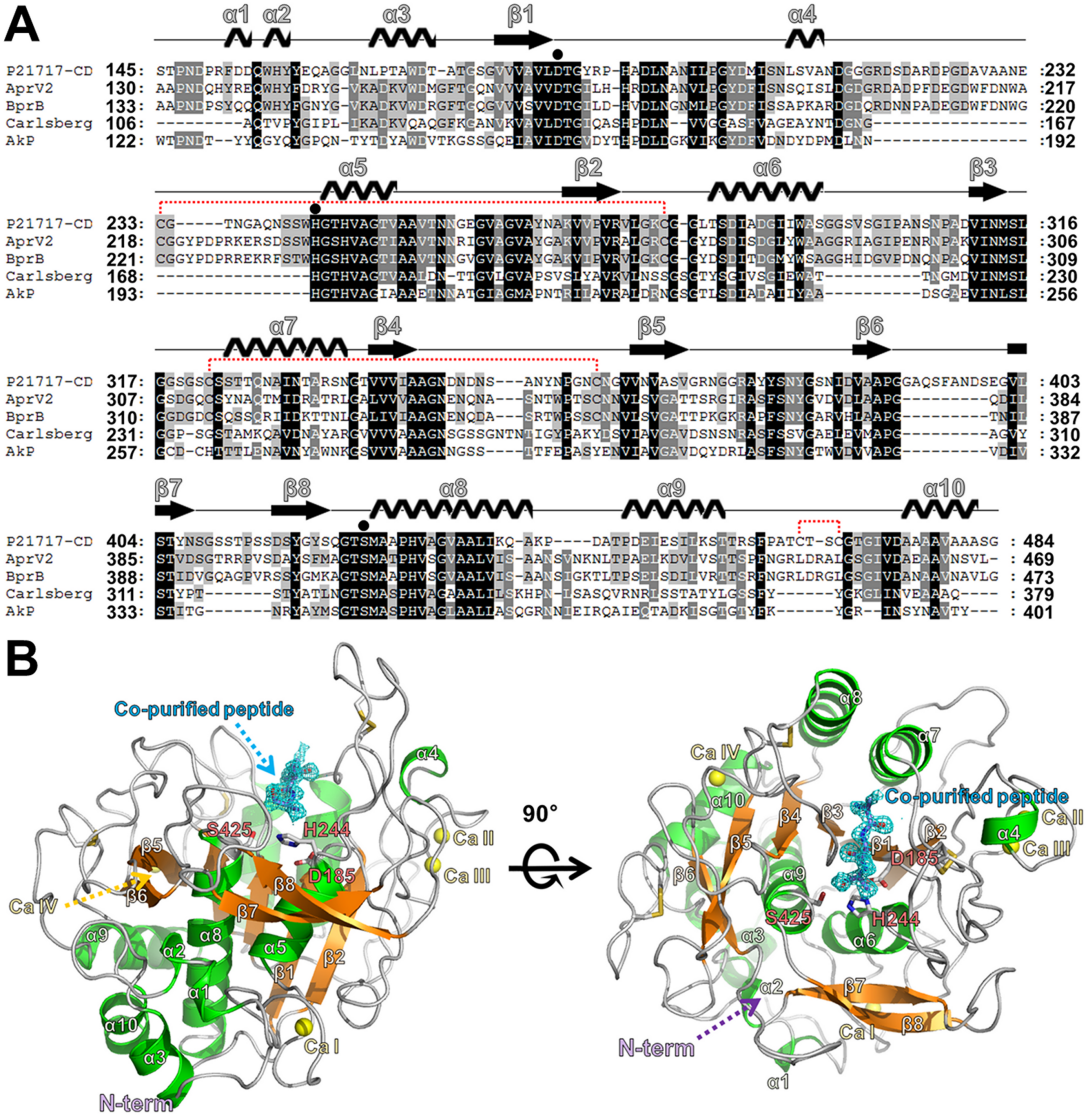
Multiple-sequence alignment and crystal structure of Pro21717-CD. (A) Structure-based alignment of the amino acid sequences of serine proteases from *Pseudoalteromonas arctica* PAMC 21717 (Pro21717), *Dichelobacter nodosus* (AprV2), *Dichelobacter nodosus* (BprB), *Bacillus licheniformis* (subtilisin Carlsberg), and *Bacillus sp*. strain AK1 (AkP), using ClustalX [29]. Residues constituting the catalytic triad (Asp185, His244, and Ser425) are indicated by filled circles. Cysteine residues (Cys207–Cys254, Cys296–Cys333, and Cys439–Cys442), joined by red-dotted lines, represent intramolecular disulfide bond formation. (B) Ribbon diagram of Pro21717-CD showing active site residues and secondary structure elements. The 2Fo–Fc electron density map (contoured at 1σ) around the co-purified peptide is shown in blue. The bound calcium ions are presented with yellow balls, and disulfide bonds are shown with yellow sticks.

**Fig 3:**
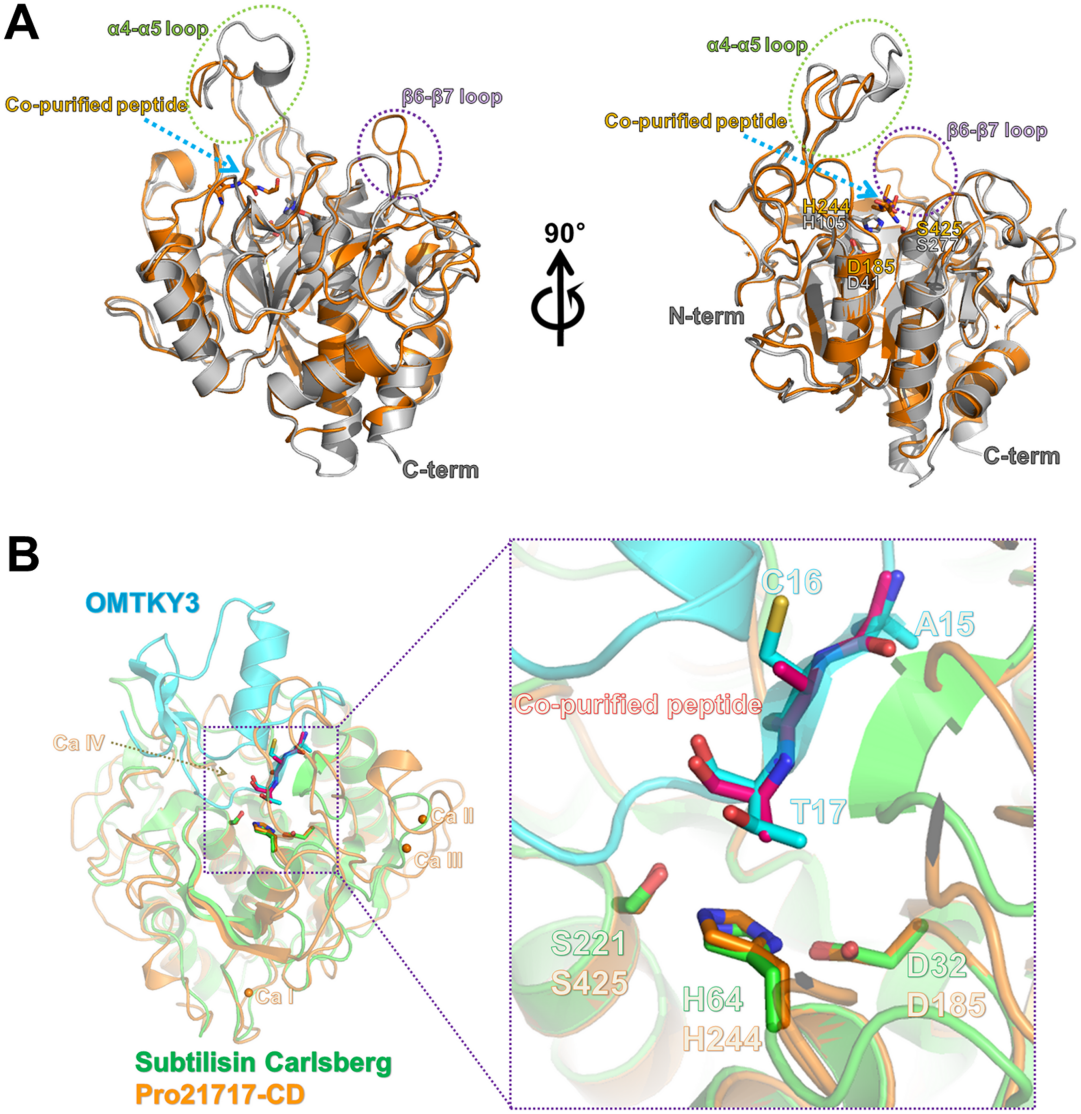
Structural comparison of Pro21717-CD with other serine proteases. (A) Superimposition of Pro21717-CD (orange color) onto AprB2 protease (an extracellular protease from *Dichelobacter nodosus*; PDB code: 3LPC; gray color) revealed that Pro21717-CD adopts a novel conformation in the capping-loop region of the active site. (B) Structural comparison of Pro21717-CD (orange) and subtilisin Carlsberg (green) complexed with the protease inhibitor OMTKY3 (cyan). Notably, the conformation of the co-purified peptide in the active site of Pro21717-CD is identical to that of the residues (Ala15[P4], Cys16[P3], and Thr17[P2]) in the subtilisin Carlsberg inhibitor (OMTKY3) (right panel).

The crystal structure of Pro21717-CD (final modeled residues 145–483; the first 115 residues of pro-sequence were cleaved from Pro21717-PCD by autocatalytic processing during the refolding step) was determined to a resolution of 1.4 Å using a molecular-replacement approach [24], based on the structure of the AprV2 protease (GenBank Accession No. CP000513; PDB code 3LPA) [25]. In the asymmetric unit, one Pro21717-CD molecule was found, which had a classical subtilisin-like fold consisting of 10 α-helices and 8 β-strands. Pro21717 contains three disulfide bonds (Cys207–Cys254, Cys296–Cys333, and Cys439–Cys442) (Fig 2A) and four calcium-binding sites that may influence protein stability. The fourth calcium-binding site is unique to Pro21717 and does not appear in other subtilisin-like proteases that have been structurally characterized to date. Notably, the AprV2 protease structure (PDB code 3LPA) contains only three Ca ions (CaI, CaII, and CaIII) in its structure located at similar positions compared with the Pro21717-CD structure. The CaIV ion in Pro21717-CD is stabilized by Asp385, Glu451, the carbonyl O atom of Val362, and two water molecules. Although the structure of eglin c bound to subtilisin Carlsberg (PDB code 1CSE) has a calcium ion near the CaIV position of Pro21717-CD, the interacting residues were different because subtilisin Carlsberg has an Arg residue in the position corresponding to Glu451 in Pro21717-CD [26]. The predicted active site containing the catalytic triad residues (Asp185, His244, and Ser425) is located at the C-terminal end of the β-sheet and forms a deep elongated pocket. The Ser—His—Asp catalytic triad adopts a similar conformation to that found in other subtilisin-like serine proteases [27]. Examination of the Fo–Fc difference density map after refinement showed additional strong and continuous density near the active site (Fig 2B). The conformation of this density could clearly be identified as a small peptide bound to the predicted substrate-binding site (S2–S3–S4). Thus, we inferred that the co-purified ligand is a small peptide substrate (or product) that could have been derived from the pro-sequence of Pro21717 during the refolding step. However, peptide sequence identification was not successful. Thus, the final model structure of Pro21717-CD contains only three alanine peptides in the substrate-binding site. Notably, the orientation of the unknown peptide in the active site of Pro21717-CD was identical to that of the interface residues (Ala15[P4]–Cys16[P3]–Thr17[P2]) within the subtilisin Carlsberg-inhibitor (OMTKY3) complex (PDB code: 1YU6) (Fig 3B) [28].

**Fig 4:**
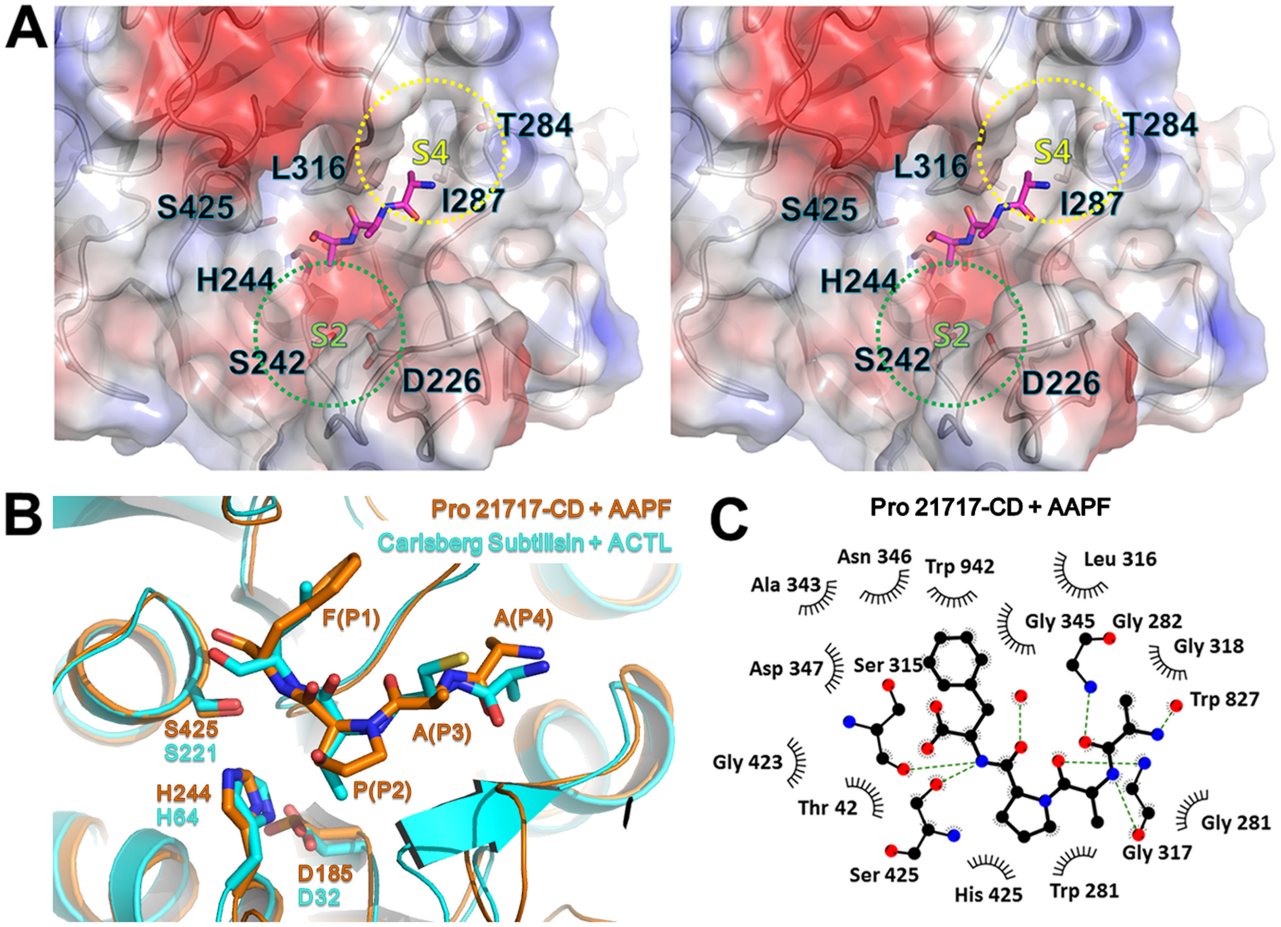
Substrate-binding mode of Pro21717-CD. (A) Stereoview of the substrate-binding site in Pro21717-CD. The bound, co-purified peptide (magenta) facilitated the characterization of the substrate-binding pocket and the substrate-binding mode of Pro21717. The side chains of the catalytic triad (Asp185, His244, and Ser425) and the residues in the nearby S2 and S4 pockets are presented as a stick model. (B) Ribbon diagram showing superposition of Pro21717-CD (orange) and Carlsberg subtilisin (cyan) at the active site. The A–A–P–F peptide in Pro21717-CD and the A–C–T–L peptide in subtilisin Carlsberg are shown as sticks. The catalytic triad residues Asp185, His244, and Ser425 (Pro21717-CD) and Asp32, His64 and Ser221 (subtilisin Carlsberg) are also shown as sticks. (C) Schematic view of the A–A–P–F peptide-binding mode in Pro21717-CD. The key hydrogen and hydrophobic interactions involved in peptide binding are presented using Ligplot.

In agreement with the sequence similarity, a DALI search [30] for structural similarity revealed that the AprB2 protease (an extracellular protease from *Dichelobacter nodosus*; PDB code: 3LPC) [25] had the highest similarity to the Pro21717-CD structure (Z-score, 58.7), followed by a thermitase and a subtilisin savinase with significant DALI Z-scores of 44.6 and 41.4, respectively. The backbone structures of Pro21717-CD and AprB2 can be superimposed with a root mean square deviation (r.m.s.d.) of 0.66 Å for 319 atom numbers of C_α_. One of the distinctive features of Pro21717-CD compared with the AprB2 protease is a loop deletion (α4–α5 loop) and a loop insertion (β6–β7 loop; residues 391–401) near the active site cleft that produces a cap structure covering the deep hydrophobic substrate-binding pocket in Pro21717-CD (Fig 3A). Structural comparison of Pro21717-CD with subtilisin Carlsberg (PDB code: 1YU6) showed that Pro21717-CD has larger S2 and S4 pocket volumes, suggesting that it can accommodate a broad range of substrates, including those with bulky side chain residues. The substrate-specificity site in the S4 pocket of Pro21717 is comprised of residues Thr284, Leu316, and Ile287. However, in the subtilisin Carlsberg structure, Tyr104 replaces the corresponding Thr284 residue, reducing the space available in the S4 pocket. The other substrate-specificity site (the S2 pocket) is formed by residues His244, Ser242, and Asp226. In contrast, the inserted Ser98–Gly102 loop of the subtilisin Carlsberg structure protrudes into the active site and reduces the space of the S2 pocket. The differences in these residues suggest that the substrate-binding cleft of Pro21717-CD is wider than that of subtilisin Carlsberg, which may affect the substrate specificity of Pro21717-CD (Fig 4A). In the Pro21717-CD structure, the side chain of the P3 alanine is oriented towards the solvent region and, thus, the S3 pocket does not exist. The S1 pocket, which is a major substrate-specificity site, was also identified in Pro21717 and was predicted to bind a model peptide with the sequence A—A–P–F. The binding of this model peptide was experimentally confirmed in our protease activity assay (Table 2) and its conformation agrees well with those of related protease–peptide complexes (Fig 4B). A phenylalanine residue (P1) was stabilized by several hydrophobic interactions in the S1 pocket, which is formed from Tyr354, Ala343, and Ile342 in Pro21717-CD, and similar hydrophobic residues (Leu126, Ala152, and Gly154) found in the subtilisin Carlsberg structure produce an S1 pocket that binds leucine (Fig 4C). Interestingly, the proline–phenylalanine sequence (141-LKPF-144) is found in the prosequence self-cleavage site of Pro21717.

**Table 2:** Proteolytic activities of Pro21717-CD on various substrates.

Substrate		Relative activity (%)	
		Pro21717-CD	Subtilisin Carlsberg
Macro molecule	Collagen	8.3±2.8	6.7±1.2
	Keratin	14.1±0.3	30.2±5.6
	Azocasein	100	100
	Skim milk	41.7±2.5	67.7±5.7
Peptide	AAV	2.4±1.4	7.9±3.9
	AAPL	41.7±0.2	57.1±10.1
	AAA	2.1±0.8	10.2±1.9
	GGF	11.2±7.4	16.2±2.1
	TLV	3.8±0.1	11.9±1.1
	AAPF	100	100
	AAVA	6.4±2.1	12.9±0.6

The structural differences found in Pro21717-CD, including the altered active site loop length and substrate pocket size, indicate a wider substrate-binding site. These changes might require less conformational changes to reach the transient acyl-enzyme intermediate, thereby helping lower the optimum temperature and the activation energy of the catalyzed reaction. Moreover, analysis of Pro21717-CD secondary structures revealed a striking increase in the loop content, compared with subtilisin Carlsberg. Indeed, the distribution of residue content between loops, helices, and strands was found to be 60.1% versus 53.0%, 26.6% versus 34.7%, and 13.3% versus 12.3%, respectively, in Pro21717-CD versus subtilisin Carlsberg. In detail, Pro21717-CD has longer loop regions compared with the Carlsberg protease due to several loop insertions in the α4–α5, α6–β3, β6–β7, β7–β8, and α9–α10 regions. These differences may also contribute to the increased backbone flexibility in Pro21717-CD, and these elements may act synergistically to allow the conformational flexibility needed for efficient catalysis in cold environments.

### Effects of temperature and pH on Pro21717-CD activity and stability

**Fig 5:**
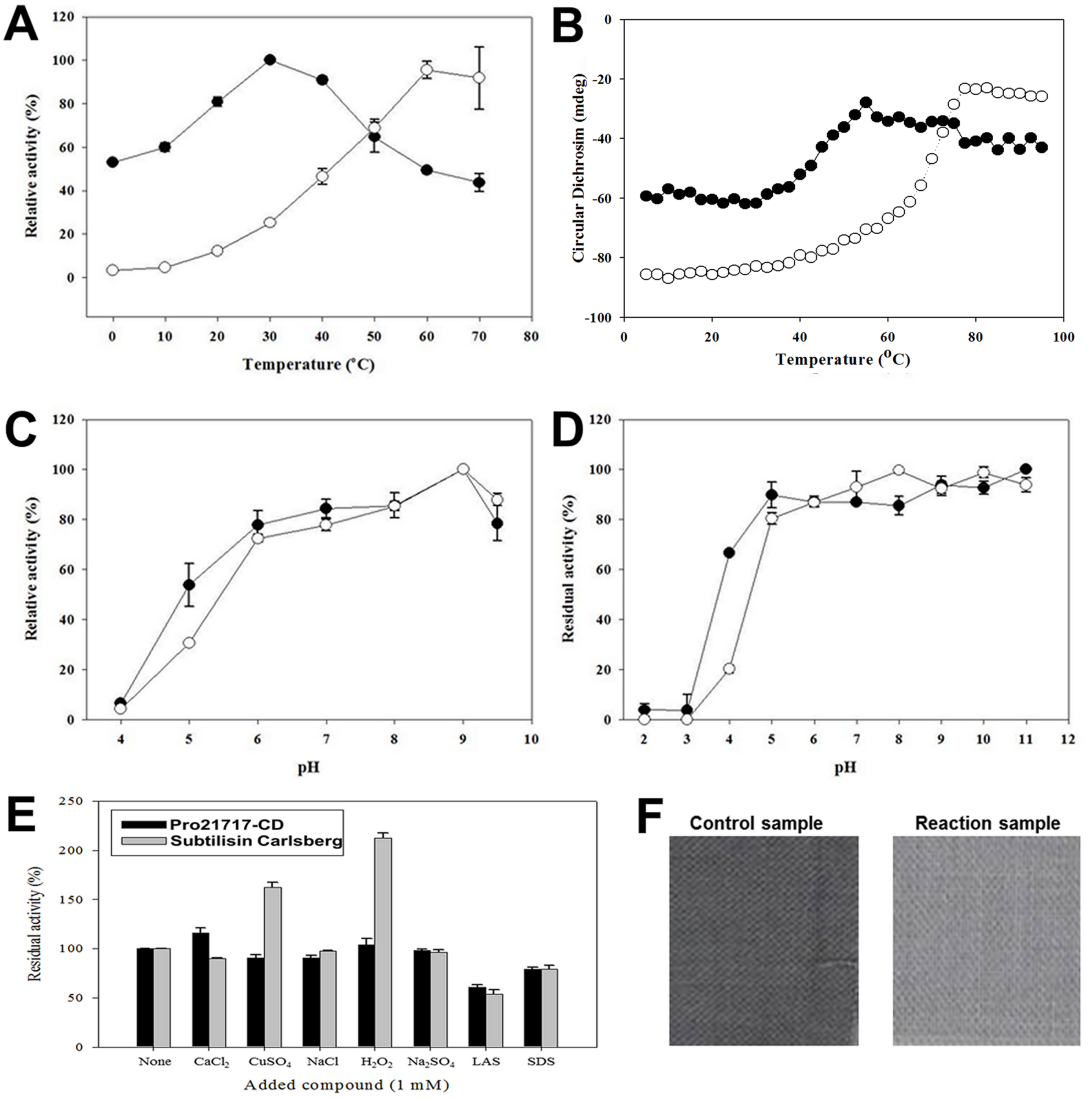
Activity and stability assays for Pro21717-CD. (A–D) Thermal stability and the effect of pH on Pro21717-CD activity (black circles), compared with those for subtilisin Carlsberg (open circles). The optimal temperature (A) and pH (C) for activity were measured, and the thermal stability of Pro21717-CD and subtilisin Carlsberg (B), and the activity in various pH values (D) were examined as described in the Materials and Methods section. Briefly, the thermal melting profile of Pro21717-CD and subtilisin Carlsberg were recorded using CD spectroscopy. The signal at 220 nm was recorded while the temperature was increased from 5 to 95°C at intervals of 2.5°C. (E) The effects of metal ions and detergents on Pro21717-CD activity and stability. (F) Effect of recombinant protease Pro21717-CD on milk/blood/ink stain removal. The washing test was performed at 15°C in LAS detergent in water (control) or LAS detergent containing 13 mg of Pro21717-CD in water (reaction).

Additive enzymes for laundry detergents, which are intended to work well in cold tap water, should have both higher activity and stability at low temperature and in alkaline pH. Thus, the activity of Pro21717-CD was analyzed at various temperatures. The proteolytic activity was highest in the range of 30–40°C, with an optimum at 30°C, but rapidly decreased at temperatures higher than 40°C (Fig 5A). Pro21717-CD exhibited relative activities of 53% (142.7 ± 2.9 U/mg) and 60% (160.8 ± 0.9 U/mg) at 0 and 10°C, respectively, compared to 100% (268.6 ± 7.5 U/mg) at 30°C. In contrast, mesophilic subtilisin Carlsberg from *Bacillus licheniformis* (Sigma–Aldrich), one of the first proteases used in detergents, showed lower activities of 3% (16.1 ± 0.5 U/mg), 5% (22.6 ± 0.8 U/mg), and 25% (124.5 ± 3.5 U/mg) at 0, 10, and 30°C, respectively, compared to 100% (471.6 ± 4.7 U/mg) at 60°C. It is very interesting that the relative activity (53%) of recombinant Pro21717-CD near 0°C was much higher than those of the wild-type psychrophilic proteases from several cold-adapted bacteria: *Colwellia psychrerythraea* 34H (12%) [6], *Colwellia* sp. NJ341 (30%) [8] and *Pseudoalteromonas* sp. SM9913 (45%) [5]. The denaturation temperatures of Pro21717-CD and subtilisin Carlsberg were calculated at 45.5°C and 67.6°C, respectively. Moreover, we confirmed that the thermal stability of Pro21717-CD was reduced at 40.0°C by adding reducing agent (1 mM β-mercaptoethanol). That result indicated that the disulfide bonds in Pro21717-CD are important for its thermal stability. Detailed CD data are provided in Fig 5B and S1 Fig. Even at low temperature (10°C), Pro21717-CD exhibited relatively high turnover rate (*k*_cat_), with an optimum at 30°C. In contrast, subtilisin Carlsberg (optimal = 70°C) showed lower turnover number at below 30°C (S1 Table). Together with the cold-temperature activity described above, the thermolability of Pro21717-CD demonstrates that it is a true psychrophilic protease.

The effect of pH was determined by pre-incubating Pro21717-CD in buffered solutions with various pH values. Pro21717-CD exhibited its maximum activity at pH 9.0 (Fig 5C), confirming the alkaline tolerance of the enzyme, and it remained stable over a broad pH range of 5.0–11.0 (Fig 5D). Similarly, alkaline subtilisin Carlsberg was active between pH 5.0 and 11.0, with maximum activity at pH 9.0, and below pH 5.0 its activity decreased significantly.

### Effects of metal ions and chemical reagents on Pro21717-CD activity and stability

The effects of various metal ions and chemical reagents that might be included as detergent components on Pro21717-CD activity were analyzed (Fig 5E). Ca^2+^ cation (1 mM), which was reported as a thermo-stabilizer for some subtilases [31], enhanced the activity of Pro21717-CD by 16%, whereas Cu^2+^ showed no effects. The activity of Pro21717-CD was very stable during a 1-h incubation in 1 mM Na_2_SO_4_, and it was also resistant to denaturation during a 1-h incubation in 1.0–5.0% H_2_O_2_. Notably, Na_2_SO_4_ and H_2_O_2_ are common ingredients in modern bleach-based detergent formulations. The detergent surfactants linear alkylbenzene sulfonate (LAS) and SDS were also tested, resulting in slightly reduced Pro21717-CD activities of 60.7% ± 2.7% and 78.9% ± 2.1%, respectively. Pro21717-CD was resistant to denaturation by Na_2_SO_4_ and H_2_O_2_, common ingredients in modern bleach-based detergent formulations [32]. To date, only a few reports are available for H_2_O_2_-stable enzymes [33]. The detergent surfactants linear alkylbenzene sulfonate (LAS) and SDS were also tested, resulting in slightly reduced Pro21717-CD activities of 60.7% ± 2.7% and 78.9% ± 2.1%, respectively. In comparison with a commercial detergent enzyme, Pro21717-CD showed resistance and stability that was similar to subtilisin Carlsberg (the activity of which was also slightly reduced by LAS and SDS), although Cu^2+^ and H_2_O_2_ enhanced the activity of subtilisin Carlsberg but not Pro21717-CD.

### Substrate specificity of Pro21717-CD

The proteolytic activity of Pro21717-CD was tested on some naturally derived proteins (collagen, keratin, azocasein, and skim milk) as well as synthetic peptides, which are typically used to evaluate the substrate specificity of proteases (Table 2). Both of Pro21717-CD and subtilisin Carlsberg showed proteolytic activity against all macromolecular proteins tested, with differing preferences for each substrate (azocasein > skim milk > keratin > collagen). Moreover, Pro21717-CD and subtilisin Carlsberg had the highest and most significant activities for AAPF and AAPL, respectively, among the small synthetic peptides tested. Overall, the substrate specificity of Pro21717-CD for various substrates was similar to that of subtilisin Carlsberg, showing activities against complex substrates, such as collagen and keratin, and a preference for the neutral amino acid proline during the cleavage of small peptides.

### Washing test with Pro21717-CD

Because proteinaceous stains generally include blood, milk, egg, and sauces [20], the washing capability of Pro21717-CD was tested with a variety of substances. The activity of Pro21717-CD on blood, milk and ink bound to cotton cloth was tested in a standard formulation for LAS detergents that was provided by a laundry detergent manufacturer. In a preliminary test, Pro21717-CD completely hydrolyzed 0.5% and 1.0% skim milk within 4 min at room temperature, significantly decreasing the turbidity. When the washing performance was tested with the test fabric, treatment with 13 mg of Pro21717-CD at 15°C for 1 h removed the stains, significantly clearing the heavily stained fabric compared to a control sample without Pro21717-CD (Fig 5F). This washing test in cold tap water shows that Pro21717-CD, with its higher stability and washing performance in detergents, appears to have strong potential for use in new laundry detergent formulations.

## Discussion

Here, we report, to our knowledge, the first cold-adapted structure of a member of the subtilisin protease family, Pro21717-CD. Notably, the only structure of a cold-active protease, namely Apa1 from *Pseudoalteromonas* sp. AS-11 [34] (PDB code 1V6C), appeared on the RCSB website (https://www.rcsb.org), but the structure has not yet been published. Although several members of the subtilisin superfamily of proteases have been identified in a variety of organisms, a large proportion of commercially available subtilisins were originally derived from and produced in *Bacillus* species, which have advantageous properties compared to other species [20]. Detergent enzyme companies have been researching new detergent proteases either through protein engineering of existing subtilisins, or by searching for new proteases from bacterial metagenomes or isolates. Maurer [20] mentioned that every year, approximately ten new wild-type subtilisins are being described in literature. Recently, the interest in better enzyme performance at low temperatures has led to renewed efforts in screening for psychrophilic subtilisins.

In this study, we cloned a gene (*pro21717*) encoding the psychrophilic subtilisin-like protease Pro21717, which was partially purified from *P*. *arctica* PAMC 21717 and characterized for its advantageous properties as a new additive enzyme for laundry detergents. We succeeded in the heterologous overproduction of the recombinant protease from an *E*. *coli* host and achieved refolding of the protein to a functional protease (Pro21717-CD) with an average recovery rate of over 22%. Notably, the purified Pro21717-CD was very stable in solution, but began to form a dimer three days after refolding (S2A and S2B Fig). We expect that this phenomenon resulted from enzymatic autolysis or further maturation. Regardless of dimerization, Pro21717-CD showed a high specific activity and exhibited a relative activity of 60% at 10°C versus 100% at the optimal temperature of 30°C. In contrast, mesophilic subtilisin Carlsberg showed a lower cold-activity of 5% at 10°C compared to 100% at its higher optimal temperature of 60°C. Polarzyme, a new low-temperature detergent enzyme from Novozymes, showed a relative activity of 35% at 10°C in comparison to 100% activity at its optimal temperature of 50°C.

The subsequent success or failure of these enzymes as commercially viable additives will depend not only on washing performance and compatibility with commercial detergent formulations, but also on their yields during fermentation. Currently, much reliable information regarding the production of recombinant enzymes in *E*. *coli* hosts is publicly available. Therefore, the composition of fermentation media and the details of the fermentation processes could likely be optimized to produce Pro21717-CD at the levels potentially required for commercialization.

Overall, the recombinant Pro21717-CD displayed significant activity at alkaline pH and was stable when exposed to detergent surfactants and other common chemical components of laundry detergents. This biochemical characterization and a successful washing test indicated that the useful properties of Pro21717-CD are comparable to those of subtilisin Carlsberg, which is being produced at a commercial level under the trademark Alcalase by Novozymes. Moreover, Pro21717-CD has higher activity at low washing temperatures than subtilisin Carlsberg does. These characteristics could reflect the wide pocket size (S3A Fig). Pro21717-CD mutants had narrower pocket size and showed low activity (76–80%) compared to the wild-type enzyme at 5°C (S3B Fig). In addition, we examined optimized concentrations of stabilizers for Pro21717-CD (S2 Table and S4A Fig). The enzyme containing stabilizing mixture maintained up to 70% proteolytic activity for 42 d at 37°C (S4B Fig). In conclusion, Pro21717-CD has the potential to be used in commercial detergent formulations as a new psychrophilic detergent enzyme, either alone or in combination with other mesophilic enzymes such as subtilisin Carlsberg.

## Supporting information

S1 Fig Thermal stability of Pro21717-CD. (PDF)

S2 Fig Homodimerization of Pro21717-CD. (PDF)

S3 Fig Effects of the substrate pocket size on Pro21717-CD activity. (PDF)

S4 Fig Improvement of Pro21717-CD stability. (PDF)

S1 Table Kinetic parameters of Pro21717-CD and subtilisin Carlsberg activity against azocasein at various temperatures. (PDF)

S2 Table Box–Behnken optimization of significant enzyme stabilizers. (PDF)
