# Supplementary material for: Crystal structure of a cold-active protease (Pro21717) from the psychrophilic bacterium, Pseudoalteromonas arctica PAMC 21717, at 1.4 Å resolution: Structural adaptations to cold and functional analysis of a laundry detergent enzyme
Source: PLoS One. 2018 Feb 21;13(2):e0191740. doi: 10.1371/journal.pone.0191740 (PMC5821440; doi:10.1371/journal.pone.0191740)
Supplement: S1 Fig — (PDF) [file pone.0191740.s001.pdf]

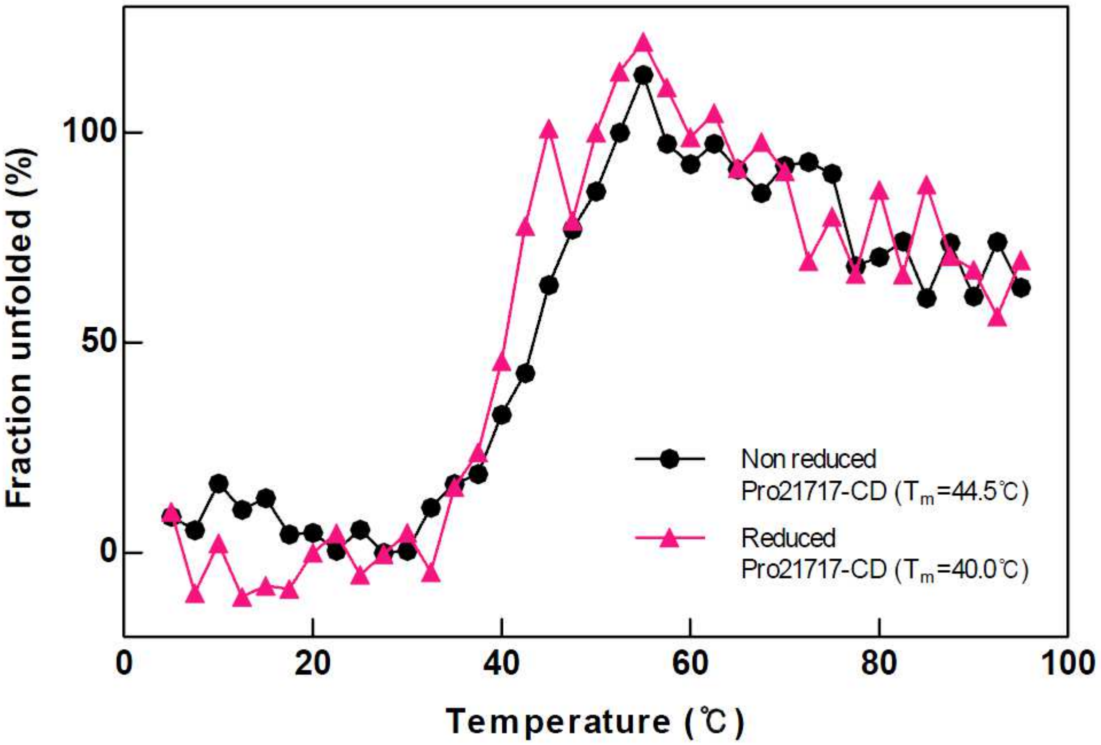

Figure S1. Thermal stability of Pro21717-CD. Thermal denaturation of non-reduced Pro21717-CD (black circle) and reduced Pro21717-CD (with 1 mM β-mercaptoethanol; magenta triangle) was measured using Circular-dichroism (CD) spectroscopy (Chirascan CD spetropolarimeter, Applied Photophysics, Surrey, UK) connected to a Peltier temperature controller. Protein samples (0.5 mg/ml) in 20 mM Tris-HCl pH 8.0, 150 mM NaCl and a 0.1 cm path length cuvette were used for spectral data collection. Changes in ellipticity were recoreded at a wavelength of 222 mm by heating the protein sample between 5 and 95°C at intervals of 2.5°C. The denaturation temperatures (T_m) were defined as the point at which 50% of the sample denatured. The effect of β-mercaptoethanol on Pro21717-CD activity was also tested. Pro21717-CD exhibited a residual activity of 83.4±7.4 or 51.8±2.6% compared to non-treated control (100%) by the addition of 1 or 10 mM β-mercaptoethanol, respectively. These results suggest that the disulfide bonds in Pro21717-CD are important for the enzymatic activity as well as its stability.
