# Supplementary material for: Crystal structure of a cold-active protease (Pro21717) from the psychrophilic bacterium, Pseudoalteromonas arctica PAMC 21717, at 1.4 Å resolution: Structural adaptations to cold and functional analysis of a laundry detergent enzyme
Source: PLoS One. 2018 Feb 21;13(2):e0191740. doi: 10.1371/journal.pone.0191740 (PMC5821440; doi:10.1371/journal.pone.0191740)
Supplement: S2 Fig — (PDF) [file pone.0191740.s002.pdf]

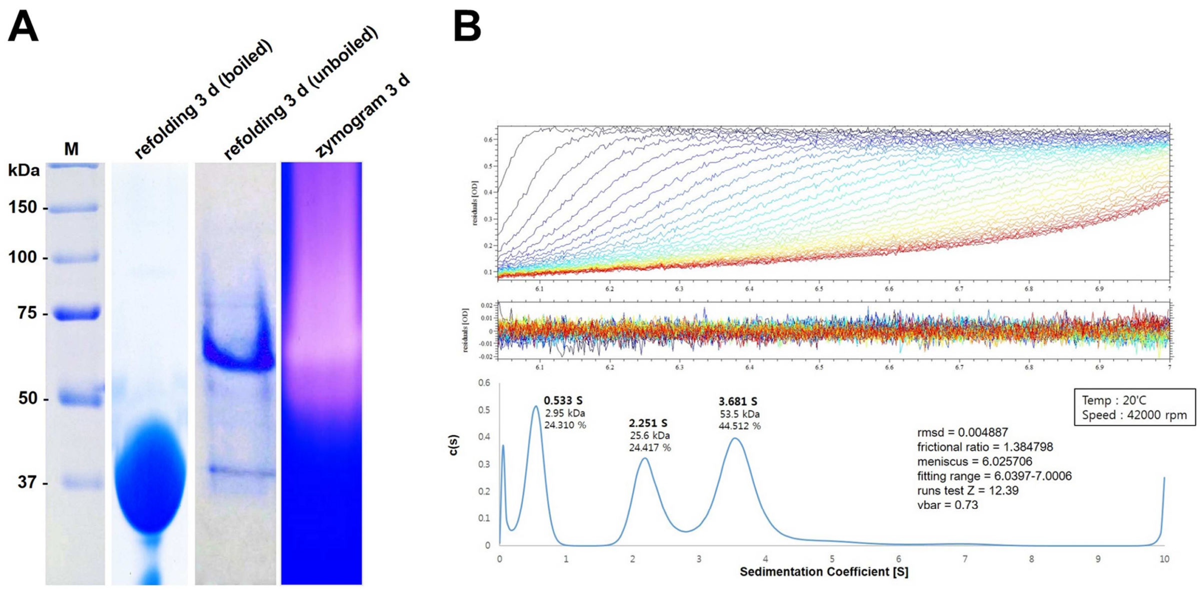

Figure S2. Homodimerization of Pro21717-CD. (A) The protein solution, refolded for 3 d, was loaded onto a 10% SDS-PAGE gel with or without 0.3% skim milk. The band for dimerized Pro21717-CD protein (not boiled, approximately 60 kDa) appeared three days later after refolding in the SDS-PAGE and zymography gels. In addition, our N-terminal sequencing data showed that the Pro21717-CD protein had two N-terminal amino acid sequences (STPNDP, starting point of catalytic domain; SMAAP, part of C-terminal Pro21717-CD fragment, molecular weight of approximate 6 kDa). That means the Pro21717-CD protein undergoes further autolysis or maturation (data not shown here). (B) Analytical ultracentrifugation (AUC) results showed that purified Pro21717-CD formed a mixture of monomers and dimers. Notably, small fragments were also observed by AUC analysis, suggesting that further autolysis or maturation of Pro21717-CD occurred. These data were in good agreement with the results shown in (A). The AUC experiment was performed at 20ºC using a XL-A analytical ultracentrifuge (Beckman Coulter). The concentration of Pro21717-CD was 2 mg/ml in 25 mM Tris-HCl (pH 8.0), 200 mM NaCl, 5 mM $MgCl_2$, and 2 mM dithiothreitol. Movement of the Pro21717-CD protein was monitored at 45,000 rpm and a wavelength of 280 nm.
