# Supplementary material for: Crystal structure of a cold-active protease (Pro21717) from the psychrophilic bacterium, Pseudoalteromonas arctica PAMC 21717, at 1.4 Å resolution: Structural adaptations to cold and functional analysis of a laundry detergent enzyme
Source: PLoS One. 2018 Feb 21;13(2):e0191740. doi: 10.1371/journal.pone.0191740 (PMC5821440; doi:10.1371/journal.pone.0191740)
Supplement: S3 Fig — (PDF) [file pone.0191740.s003.pdf]

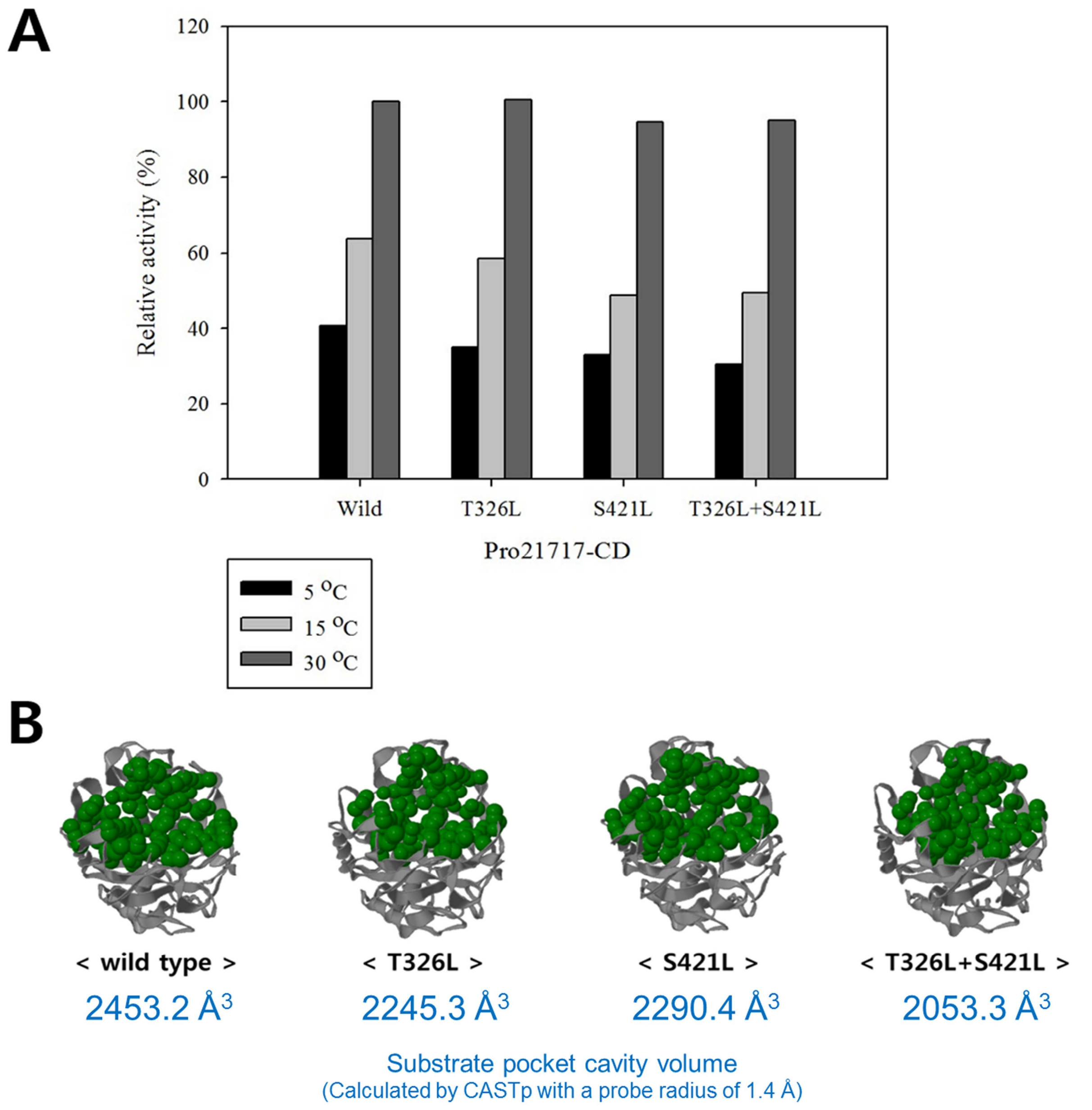

Figure S3. Effects of the substrate pocket size on Pro21717-CD activity. Three Pro21717-CD mutants (T326L, S421L, and T326L + S421L) were constructed by PCR-based mutagenesis. Theses mutants were selected based on a sequence alignment between Pro21717-CD and mesophilic subtilisin Carlsberg. It should be noted that Pro21717-CD has higher proteolytic activity than subtilisin Carlsberg at low temperatures. The following primers pairs were used to generate the T326L and S421L mutants: T326L mutant, T326L-F (5′-CGGTTCATGTAGCTCTACACTTCAAAATGCAAT-3′) and T326L-R (5′-

ATTGCATTTTGAAGTGTAGAGCTACATGAACCG-3′); S421L, S421L-F (5′-GACAGCTATGGCTACTTACAAGGTACATC-3′) and S421L-R (5′-GATGTACCTTGTAAGTAGCCATAGCTGTC-3′). A double-point mutant (T326L + S421L) was constructed using these primers pairs, sequentially. PCR was performed using PRIME STAR® HS DNA polymerase (TaKaRa) as follows: an initial denaturing step at 94ºC for 5 min, 17 cycles of amplification (94ºC for 45 s, 62ºC for 45 s, and 72ºC for 8 min), and a final extension at 72ºC for 10 min. The PCR product was treated with *Dpn*I, and mutated plasmid DNA was transferred into *E. coli* BL21 (DE3) cells. The mutant proteins were produced by the same method used to produce Pro21717-CD. (A) Proteolytic activities of Pro21717-CD and its mutants were determined at different temperatures (5, 15, and 30ºC). The activities of the wild-type and mutant enzymes were highest at 30ºC. Mutant enzymes (T326L, S421L, and T326L + S421L) exhibited activities of 101, 95, and 95% at 30ºC, respectively, relative to the wild-type enzyme (100%, 1,406 U/mg). The relative activity (41%) of the wild-type enzyme at 5ºC was higher than those of the mutant enzymes: T326L (34%), S421L (33%), and T326L + S421L (31%). (B) Three-dimensional model structures of mutants (T326L, S421L, and T326L + S421L) were built based on wild-type Pro21717-CD, using the SWISS-MODEL Repository Automated Homology Modelling database [2]. Identification and calculation of the pocket size in the crystal structures of wild-type and mutants were performed using the CASTp server [1]. These results suggested that Pro21717-CD may have cold-activity, based on its wide pocket size.

1. Dundas J, Ouyang Z, Tseng J, Binkowski A, Turpaz Y, Liang J. CASTp: computed atlas of surface topography of proteins with structural and topographical mapping of functionally annotated residues. Nucleic Acids Res. 2006;34: W116-W118.

2. Kopp J, Schwede T. The SWISS-MODEL Repository of annotated three-dimensional protein structure homology models. Nucleic Acids Res. 2004;32: D230-D234.
