# Supplementary material for: Crystal structure of a cold-active protease (Pro21717) from the psychrophilic bacterium, Pseudoalteromonas arctica PAMC 21717, at 1.4 Å resolution: Structural adaptations to cold and functional analysis of a laundry detergent enzyme
Source: PLoS One. 2018 Feb 21;13(2):e0191740. doi: 10.1371/journal.pone.0191740 (PMC5821440; doi:10.1371/journal.pone.0191740)
Supplement: S4 Fig — (PDF) [file pone.0191740.s004.pdf]

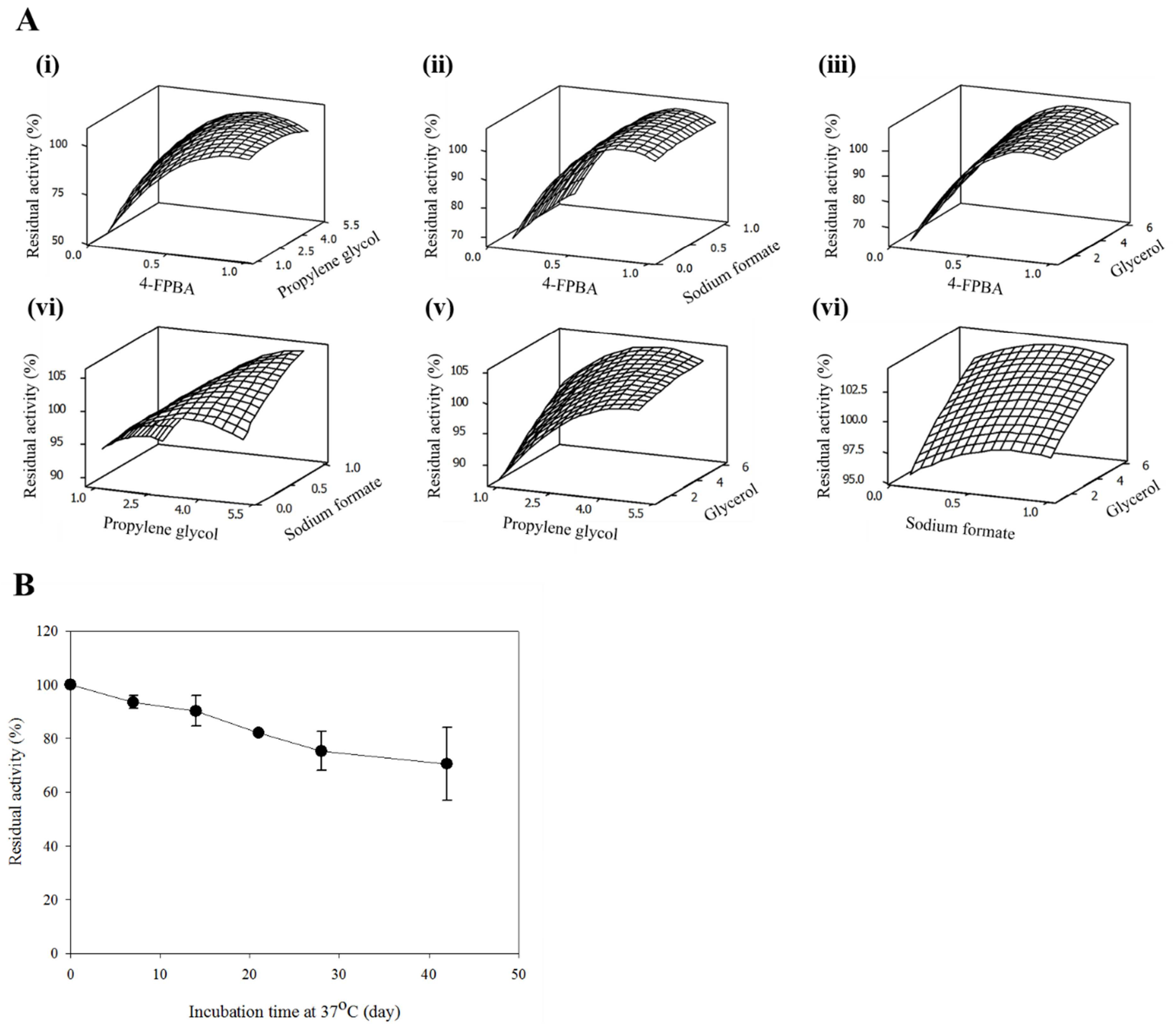

Figure S4. Improvement of Pro21717-CD stability. (A) Three-dimensional response plot displaying the effect of stabilizers ([i]–[vi]) on Pro21717-CD stability. In addition, the stabilizers were selected based on the Novozymes guidelines [1]. The enzyme stabilizer for Pro21717-CD was optimized by the Box–Behnken design (Table S1). The proteolytic activity observed in each run after a 7-d incubation at 37ºC was compared to the activity observed without thermal treating. The optimal stabilizer concentration was predicted using a second-order equation: Y (residual activity after a 7-d incubation at 37ºC) = $\beta_0 + \Sigma\beta_i X_i + \Sigma\beta_{ij} X_i X_j + \Sigma\beta_{ii} X_i^2$. The predicted Y, calculated using Minitab software (ver. 14, Minitab), was 101% when the concentrations of 4-FPBA, propylene glycol, sodium formate, and glycerol were 0.5 mM, 3.3, 0.1, and 6%, respectively. (B) Thermal stability of a Pro21717-CD–stabilizer

mixture. The stabilizer component, optimized in the Minitab program, was added to the Pro21717-CD solution, after which the mixture was incubated at 37ºC. The proteolytic activity of the mixture remained over 70% during a 42-d incubation. In contrast, the Pro21717-CD activity rapidly decreased within 1 h at 20ºC in the absence of stabilizer.

1. Bjerre J, Simonsen O, Vind J. Detergent enzymes – from discovery to product. Household and Personal Care Today. 2013;8: 37-41.
