# Supplementary material for: Crystal structure of a cold-active protease (Pro21717) from the psychrophilic bacterium, Pseudoalteromonas arctica PAMC 21717, at 1.4 Å resolution: Structural adaptations to cold and functional analysis of a laundry detergent enzyme
Source: PLoS One. 2018 Feb 21;13(2):e0191740. doi: 10.1371/journal.pone.0191740 (PMC5821440; doi:10.1371/journal.pone.0191740)
Supplement: S1 Table — (PDF) [file pone.0191740.s005.pdf]

Table S1. Kinetic parameters of Pro21717-CD and subtilisin Carlsberg activity against azocasein at various temperatures

Enzyme	Temperature	V_{max} (U/mg)	k_{cat} (sec^{-1})	K_m (%, *w*/*v*)
Pro21717-CD	10°C	1.83±0.17	1.04±0.09	0.65±0.15
	30°C	2.78±0.39	1.58±0.22	0.50±0.00
	50°C	2.17±1.17	1.23±0.66	1.90±1.10
	70°C	1.67±2.36	0.94±1.34	10.08±14.79
Subtilisin Carlsberg	10°C	0.31±0.01	0.14±0.01	0.02±0.01
	30°C	1.11±0.00	0.50±0.00	0.11±0.00
	50°C	2.78±0.39	1.25±0.18	0.24±0.06
	70°C	5.00±0.00	2.25±0.00	0.50±0.00

V_{max}, maximal velocity (1 U/mg = 1 μmole of tyrosine equivalents released/ min/ mg of protein); k_{cat}, turnover number; K_m, Michaelis constant

For enzyme kinetics experiment, 15 μg of enzymes (Pro21717-CD or subtilisin Carlsberg) was added to 1.09 mL of standard buffer containing 0.11-0.47% of azocasein. Following incubation of the reaction mixture at various temperatures (10-70°C) for 3 min, all kinetic parameters (V_{max}, k_{cat}, and K_m) was calculated by Lineweaver-Burk plot, modifying Michaelis-Menten equation.
