# Supplementary material for: Crystal structure of a cold-active protease (Pro21717) from the psychrophilic bacterium, Pseudoalteromonas arctica PAMC 21717, at 1.4 Å resolution: Structural adaptations to cold and functional analysis of a laundry detergent enzyme
Source: PLoS One. 2018 Feb 21;13(2):e0191740. doi: 10.1371/journal.pone.0191740 (PMC5821440; doi:10.1371/journal.pone.0191740)
Supplement: S2 Table — (PDF) [file pone.0191740.s006.pdf]

Table S2. Box–Behnken optimization of significant enzyme stabilizers

Variable	Stabilizer	− Value	0 Value	+ Value
X_1 (mM)	[a]4-FPBA	0.10	0.55	1.00
X_2 (%, *v/v*)	Propylene glycol	1.00	3.00	5.00
X_3 (%, *w/v*)	Sodium formate	0.10	0.55	1.00
X_4 (%, *v/v*)	Glycerol	1.00	3.50	6.00

[a]4-FPBA, 4-formyl phenyl boronic acid
